# *Cis*-motifs upstream of the transcription and translation initiation sites are effectively revealed by their positional disequilibrium in eukaryote genomes using frequency distribution curves

**DOI:** 10.1186/1471-2105-7-522

**Published:** 2006-11-30

**Authors:** Kenneth W Berendzen, Kurt Stüber, Klaus Harter, Dierk Wanke

**Affiliations:** 1 Max-Planck Institute for Plant Breeding Research, Carl-von-Linné-Weg 10, D-50829 Köln, Germany; 2 Universität Tübingen, ZMBP Pflanzenphysiologie, Auf der Morgenstelle 1, D-72076 Tübingen, Germany

## Abstract

**Background:**

The discovery of cis-regulatory motifs still remains a challenging task even though the number of sequenced genomes is constantly growing. Computational analyses using pattern search algorithms have been valuable in phylogenetic footprinting approaches as have expression profile experiments to predict co-occurring motifs. Surprisingly little is known about the nature of *cis*-regulatory element (CRE) distribution in promoters.

**Results:**

In this paper we used the Motif Mapper open-source collection of visual basic scripts for the analysis of motifs in any aligned set of DNA sequences. We focused on promoter motif distribution curves to identify positional over-representation of DNA motifs. Using differentially aligned datasets from the model species *Arabidopsis thaliana*, *Caenorhabditis *elegans, *Drosophila melanogaster *and *Saccharomyces cerevisiae*, we convincingly demonstrated the importance of the position and orientation for motif discovery. Analysis with known CREs and all possible hexanucleotides showed that some functional elements gather close to the transcription and translation initiation sites and that elements other than the TATA-box motif are conserved between eukaryote promoters. While a high background frequency usually decreases the effectiveness of such an enumerative investigation, we improved our analysis by conducting motif distribution maps using large datasets.

**Conclusion:**

This is the first study to reveal positional over-representation of CREs and promoter motifs in a cross-species approach. CREs and motifs shared between eukaryotic promoters support the observation that an eukaryotic promoter structure has been conserved throughout evolutionary time. Furthermore, with the information on positional enrichment of a motif or a known functional CRE, it is possible to get a more detailed insight into where an element appears to function. This in turn might accelerate the in depth examination of known and yet unknown *cis*-regulatory sequences in the laboratory.

## Background

The enormous amount of sequence data produced by various sequencing projects requires the development of suitable bioinformatic tools to help with functional characterization and annotation processes. One major problem in the analysis of non-coding DNA is the accurate assignment of functional attributes to defined stretches of sequence. For every gene, the sites for the initiation of transcription or translation are somehow encrypted in the DNA-sequence. Patterns of motifs define the positioning and the orientation of the basal transcription apparatus, which makes contact with the DNA at a certain position and is constituted by the RNA polymerase II complex and additional transcription factors. The DNA-stretch essential for the recruitment of this multiproteincomplex is known to be the core promoter, a region that covers some hundred base-pairs flanking the transcription start site (TSS).

Accordingly, the sequences 5' of the translation start codon ATG must encode for similar regulatory motifs that influence mRNA-abundance prior to, during and after transcription. As RNA elements embedded in the 5' untranslated regions (UTR) of the mRNA are much more complicated to study, only a few have been thoroughly characterized [1]. It is assumed that these motifs are the target sites for transcription factors or other regulatory proteins that directly or indirectly affect RNA-transcript or protein levels.

Although there are many cases known where genes remained conserved throughout organism kingdoms, only the TATA-box is mainly known to constitute a conserved motif in 30 – 40 % of the core promoters of most species [2,3]. Besides the TATA-box, our knowledge and understanding of *cis*-regulatory elements (CRE) in different organisms is sparse and in most cases CRE databases – if they exist at all – are specific to one organism. In contrast to the accessibility of sequence information via computational means, the possibilities for promoter analysis generally lag behind. Many computational programs exist that investigate promoter sequences either based on the frequency or on a positional bias [4-10]. In most cases proof of the functionality or importance of the identified motifs, in terms of a regulatory effect on transcript abundance, is lacking. The identification of sequence patterns involved in regulation is therefore an ongoing challenge for the in-depth understanding of gene expression.

Promising work has been done in the plant field with *Arabidopsis thaliana*, where the genome sequence is completely available, the amount of non-coding sequences is small, many regions close to the TSS are annotated and large CRE databases exist [11-14,53]. Additionally, in many genomes, particularly in eukaryotes, the majority of the sequences apart from the coding region do not provide useful biological information. Some improvement in the identification of regulatory motifs was gained by combining phylogenetic data with intensive expression profile analysis of co-regulated genes [15,16,3]. This approach is not universally applicable as either promoter sequence information on close phylogenetic relatives or the availability of expression data is lacking in many cases.

Large international sequencing programs have made the genome sequences for numerous prokaryotes and various eukaryote species available. Despite the fact that the number of correctly annotated genes is increasing [51], the amount of information is not yet sufficient to predict the TSS or the promoter for most of these genomes [13,17,18]. However, TSS prediction has been optimized up to 70% accuracy for human genes [19].

Here, we describe an enumerative approach for the identification of functional CREs via motif distribution maps in TSS or ATG rooted datasets. Moreover, we show that motif distribution maps constitute a universal means for the identification and characterization of motifs in promoter sequences of the model organisms *Arabidopsis thaliana*, *Caenorhabditis elegans*, *Drosophila melanogaster *and *Saccharomyces cerevisiae*. Our analysis identifies several motifs present in the promoters of all these eukaryote species, implying a universal promoter architecture that extends over various kingdoms and goes beyond our present image of promoter conservation.

The quality of our automated bulk extraction of promoters rooted with the TSS or the ATG was initially tested with known elements that are specific to each of the sets. Subsequently, novel promoter specific motifs have been discovered. To investigate previously known CREs, motif distribution maps were constructed, showing specific curve characteristics such as maxima and minima. Here, we focused on the analysis of conserved maxima only. Our results also support the quality of the genome sequences as such, and demonstrate that these are sufficient for bulk analysis of a large number of CREs.

## Results and discussion

### Comparison of tetranucleotide sequences

There are several recent publications on the identification of putative *cis*-regulatory elements based on a frequency bias or on the position of a certain DNA-motif [4,17,7-10]. We decided to combine both methods by aligning sequences with respect to the annotated transcriptional start site (TSS) or the ATG and search for motifs that have alterations of both frequency and position. The motifs under investigation were plotted in respect to their position to form motif distribution maps, which were subsequently analyzed for positional over- or under-representation. A functional motif is defined by a frequency disequilibrium; a non random distribution at a precise position [11,17,54]. As a similar approach was undertaken earlier by Arkhipova with a preliminary, hand edited dataset from *Drosophila melanogaster *[20], we first attempted to reproduce her observations by using our script collection and the annotated genomic sequence available at the NCBI.

This analysis was performed on raw counts, but we have decided to display all graphs for reasons of comparability, as the percent of motifs relative to the total amount of sequences per dataset.

The distribution of the tetranucleotide sequences TATA and TCAG, which represent the palindromic TATA-box core and the cap-site initiator motif respectively, was analyzed by Arkhipova [20] in 252 *Drosophila *derived TSS rooted sequences spanning -250 bp to +50 bp. On the basis of her work, Ohler et al. [21] determined the element distribution first in a frequency and subsequently in a position dependent manner in 1941 sequences using the same TSS region.

Here, we were able to extract 7954 *Drosophila *sequences surrounding the annotated TSS from -250 bp to +50 bp. To get an insight into how well the TSS's are annotated in the databases, we used the 1941 sequences from Ohler et al. [21], whose accuracy was validated using BLAST searches [22].

**Figure 1:**
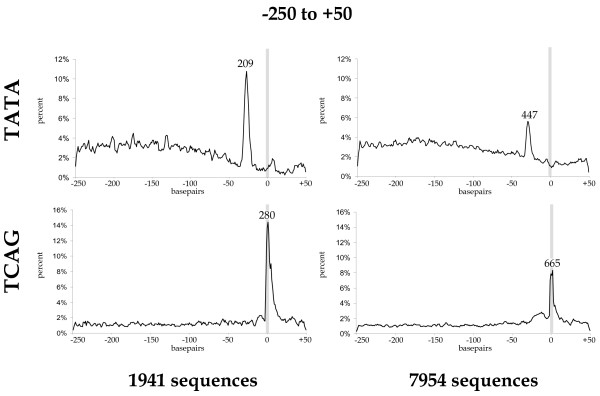
**Comparison of the motif distribution curves for the TCAG and TATA motifs in *Drosophila *promoters**. Previously examined motifs, TCAG and TATA [20], were mapped in a hand edited dataset constructed by Ohler et al. [21] containing 1941 promoter sequences and an automatically assembled dataset of 7954 sequences derived from NCBI [59]. Relative number of motifs per site (in percent) was mapped to their respective position. Datasets spanning -250 bp to +50 bp were rooted with the position of the transcription start site (TSS) indicated as a grey bar. The absolute number of aligned sequences contributing to the maximum raw value of the peaks is given above the curves [see Additional File 7].

Figure 1 shows motif distribution maps for TATA and TCAG derived from our dataset compared with the 1941 high quality, hand edited sequence set by Ohler et al. [21]. As expected, the TATA-motif exhibits its highest frequency at about -28 bp, while the TCAG-motif is found to be relatively enriched exactly at the transcription initiation site. The observed background frequencies of the motifs in the two datasets are of comparable percentages, whereas the relative peak height for the motifs of our GenBank dataset is about half of the Ohler et al. [21] dataset (Figure 1). The small portion of promoters containing a TATA-box and the conservation of its relative position coincide with previous works [23,21,20].

### Positional disequilibria of functional regulatory motifs

To get an impression of how much a motif is over-represented, we initially averaged over all counts per nucleotide position and calculated the standard deviation for the -250 bp to +50 datasets. We considered a peak to be significant if it deviated by four or more standard deviations from the background average. Additionally, to limit the computational work, we focused on the highest peak for each motif only.

For the dataset -250 bp to +50 bp rooted to the TSS we had to assume that coding sequences might influence this dataset, as 5'UTRs are sometimes shorter than 50 bp. To find out how specific the positional information on the four tetranucleotide sequences TATA, ATAA, TCAG and TCAT is with respect to the TSS, we analyzed their curve profiles in six different datasets (Table 1): two -250 bp to +50 bp datasets and four additional sets covering longer upstream regions -1500 bp to +50 bp and -1500 bp to -1 bp, all of which were rooted to the TSS or the ATG.

**Table 1:** Evaluation of tetranucleotide sequences for positional disequilibria in different *Drosophila *promoter datasets.

Dataset	Element	Highest Peak (location)	*SD *above	BG-average	*SD*
Ohler et al. [21]	ATAA	220 (-29)	5.3	65.2	29.4
	CATG	no peak	-	18.7	4.8
	TATA	209 (-30)	5.8	49.7	27.6
	TCAG	280 (-2)	7.6	31.3	32.7
	TCAT	104 (-1)	6.6	28.9	11.3
					

TSS -250..+50	ATAA	no peak	-	279.7	77.4
	CATG	no peak	-	73.5	13.3
	TATA	no peak	-	213.3	69.7
	TCAG	665 (-1)	7.7	115.5	71.6
	TCAT	268 (-1)	7.1	118.8	20.9
					

ATG -250..+50	ATAA	no peak	-	322.4	102.4
	CATG	2806 (-1)	8.5	124.8	315.9
	TATA	no peak	-	224.6	79.8
	TCAG	no peak	-	157.0	31.8
	TCAT	628 (-2)	8.7	157.8	54.1
					

TSS -1500..+50	ATAA	466 (-31)	17.6	196.2	15.3
	CATG	no peak	-	105.7	10.6
	TATA	447 (-33)	19.5	157.9	14.9
	TCAG	665 (-1)	55.1	103.6	10.2
	TCAT	268 (-1)	12.5	125.5	11.4
					

ATG -1500..+50	ATAA	567 (-3)	12.0	285.3	23.5
	CATG	2806 (-1)	204.1	136.2	13.1
	TATA	357 (-264)	6.1	227.8	21.0
	TCAG	243 (-5)	9.4	137.4	11.2
	TCAT	628 (-2)	34.9	169.1	13.1
					

TSS -1500..-1	ATAA	466 (-26)	17.6	196.1	15.3
	CATG	no peak	-	105.7	10.6
	TATA	447 (-29)	19.6	157.9	14.8
	TCAG	226 (-21)	12.0	103.6	10.2
	TCAT	174 (-1041)	4.2	125.5	11.5
					

ATG -1500..-1	ATAA	469 (-196)	7.8	285.2	23.5
	CATG	no peak	-	136.2	13.1
	TATA	357 (-263)	6.2	227.7	21.0
	TCAG	243 (-4)	9.4	137.4	11.2
	TCAT	no peak	-	169.1	13.1

The hand edited dataset from Ohler et al. [21] was compared with datasets based on annotated promoters that were extracted from genomic sequences derived from the NCBI [59]. The evaluation was conducted using motif distribution curves. Based on previous analysis [20], the significance cutoff frequency for a positional disequilibrium must differ by ≥ 4 *SD *above the overall background average [see Additional File 8]. *no peak*: based on our evaluation, no significant peaks could be identified.

As we wanted to exclude the TCAT-motif from overlapping with the ATG translation start codon in the ATG rooted datasets, we also included the CATG-motif in our analysis for comparison. If the TCAT motif were a part of the translation start site, we would expect to detect a peak right next to the ATG. The results in Table 1 demonstrate that the CATG-motif does not form a significant peak in any of the TSS rooted datasets, however TCAT does, which is indicative for the quality of the datasets' assembly with respect to the TSS. We are therefore confident that any short 5'UTR did not cause an informative peak detected in the TSS dataset.

Moreover, a significant overrepresentation of ATAA, TATA or TCAG was confirmed for the dataset used by Ohler et al. [21]. Under our strong confidence interval neither the palindomic TATA-motif nor the ATAA-sequence was considered to have a significant peak in both small -250 bp to +50 bp GenBank assembled datasets. This can be explained by the lower peak height in our datasets derived from GenBank (Table 1). Therefore, we computed a different background model using counts from -1494 bp to -494 bp for each of the larger datasets (i.e. ≥ 1500 bp; Table 1). This reduced the background noise and we could prove that all four motifs investigated by Arkhipova [20] and Ohler et al. [21] are significantly enriched in all of the larger GenBank datasets. Remarkably, the TCAG/T element again generally marks the TSS while the TATA-box core is found about -30 bp upstream (Table 1).

The two related tetranucleotides, CATG which is predominantly found to constitute the translation start site and the TCAT-transcription initiation motif, carry the common trinucleotide CAT and can principally represent two different, non-overlapping molecular functions.

This hypothesis was resolved, when the translation start codon ATG was included in any of the sets, the CATG-motif was indeed the most prominent and overlapped with the position of the translation start site (Table 1). On the other hand, the CATG-motif did not have a peak in any TSS rooted dataset. Finally, if the translation start codon ATG was removed from the analysis in the large ATG rooted datasets of -1500 bp to -1 bp, the CATG-motif was no longer significantly present. We concluded that we were able to differentiate clearly between similar motifs even with partially overlapping sequences. Hence, these similar motifs may encode different functions with respect to their position.

### Evolutionary conservation of the Inr motif

An interesting question was, whether it might be possible to differentiate between the known functional TSS consensus (*Inr*) of *Drosophila *TCAKTY [24] and the more degenerate mammalian YYANWYY *cis*-regulatory element [25]. To address this question, we conducted a comparative motif analysis on our two *D. melanogaster *GenBank datasets spanning -1500 bp to +50 bp with these two motifs.

**Figure 2:**
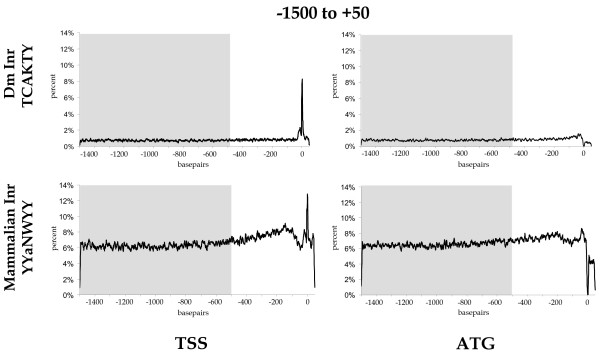
**Distribution curves of the transcription initiation motifs from mammals and *Drosophila *in promoter datasets of *Drosophila***. The motif distribution curves of the transcription initiation motif (*Inr*) of *Drosophila *TCAKTY [24] and of mammals YYANWYY [25] were constructed on 7954 automatically assembled sequences derived from NCBI [59]. Relative number of motifs per site (in percent) was mapped to their respective position. The grey box indicates the region used to calculate the background average and its *SD*.

Mapping the TCAKTY motif in the 7954 Drosophila sequences rooted to the TSS, we found a distinct positional peak at the transcription initiation site (Figure 2). Interestingly, we could also identify a significant peak for the degenerate mammalian pattern YYANWYY in the Drosophila promoter sequences at about the same position. Although it was more frequent due to its degeneracy, the peak for the mammalian pattern YYANWYY still exceeds our strong confidence interval of 4 times the standard deviation over the background average. When a similar dataset of 10672 sequences aligned to the start codon ATG was used, both motifs lack significant curve profiles suggesting positional enrichment at the transcription start site only (Figure 2).

Our observations support the overall correctness of the annotated GenBank sequence data available, reaffirm that the specific Drosophila TSS is well characterized and illustrate a generally conserved nature of eukaryotic transcription information, since the mammalian TSS does yield a significant peak at the Drosophila TSS.

### Motif distribution curves on the TATA-box

Although recent publications claimed that the annotated sequences at the primary DNA-databases were not good enough for unbiased bulk analysis of the TSS-upstream sequences [13], we concluded the opposite on the basis of the data presented so far. Hence, we broadened our analysis and acquired the sequences upstream of the TSS or the ATG for the model organisms *Arabidopsis thaliana*, *Caenorhabditis elegans *and *Saccharomyces cerevisiae *that are representatives of three organism kingdoms. As there were only very few genes in yeast that had an annotated TSS, we focused on the dataset aligned to the translation start codon ATG for this species only. As the most studied eukaryotic CRE is the TATA-box, we concentrated on the TATA-box hexanucleotides TATAAA and TATATA, as these motifs appear to form the functional TATA-box sequences at least for *Drosophila *and *Arabidopsis *[11,26,21,14].

**Figure 3:**
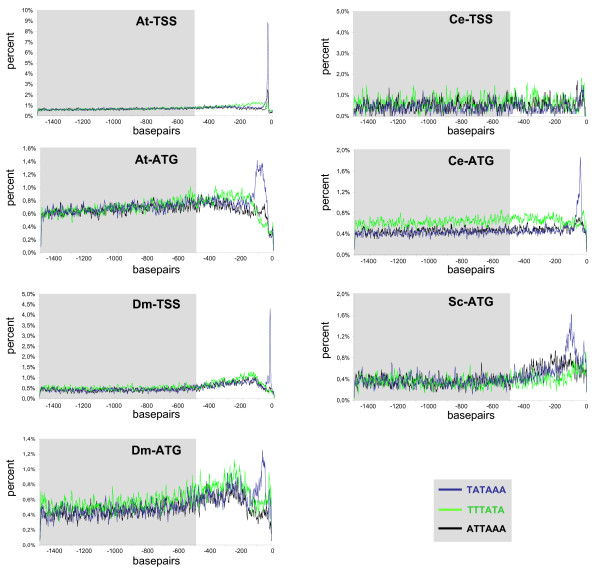
**Distribution curves of the TATA-box motif TATAAA, its antisense TTTATA and the T/A-rich motif ATTAAA**. The motif distribution curves of the TATA-box hexanucleotide TATAAA, its antisense TTTATA and the T/A-rich motif ATTAAA were constructed on automatically assembled datasets of the *Arabidopsis*, *Caenorhabditis*, *Drosophila *and *Saccharomyces *genome sequences. Relative number of motifs per site (in percent) was mapped to their respective position. The grey box indicates the region used to calculate the background average and its *SD*.

To our knowledge no one has so far compared the positional occurrence of TATA-box sequences in different organisms. We expected no distinct disequilibria for both TATA-box motifs in the ATG rooted datasets for all four organisms; instead, we assumed that there would be a blurring effect caused by varying 5' UTR lengths. But the TATAAA motif formed clearly visible positional peaks in all of the datasets in the expected location (Figure 3), which was in agreement with other reports on TATA-box function [13,26,24,23]. The TATA-box motifs in S. cerevisiae have a wider breadth of enrichment and a double peak even though they are rooted to the ATG and not the TSS (Figure 3, see Additional File 1). This may be explained by the known fact that the TATA-box motif has a different positional dependence in S. cerevisiae (-120 bp to -45 bp instead of -30 bp; [57]). Moreover, its relative distance to the ATG was comparable between the different organisms and, hence, the average size of the UTRs must be similar. Indeed, when we plotted the length distribution of the UTRs of *Arabidopsis thaliana*, *Caenorhabditis elegans *and *Drosophila *melanogaster, the UTRs of plant and the fly were similar in size, whereas the average *C. elegans *UTR was much shorter [see Additional File 2]. *Saccharomyces *is reported to have its average TSS at -15 bp to -75 bp before the ATG [23].

One important point is that the random frequency variation within the motif distribution maps decreases with the number of total sequences per dataset, which consequently affects the background based on the standard deviation. Therefore, the large *Arabidopsis thaliana *ATG rooted dataset with 26299 sequences was the most suitable to work with, while, in contrast, the *C. elegans *TSS rooted dataset with only 885 Sequences generated the largest amount of background noise.

To investigate the significance of the TATAAA motif, distribution curves were conducted for its antisense sequence TTTATA and a motif having the same A/T-composition, ATTAAA. As depicted in Figure 3, a subtle, non-significant peak could be seen for the TTTATA, even though it contains a TATA-signature and partially overlaps with the TATA-box. Furthermore, no significant maxima could be identified for the ATTAAA motif either. Thus, only the sense oriented TATAAA motif exhibits appositional disequilibrium.

The motif distribution maps conducted on the TSS aligned datasets revealed distinct peaks for TATA-box hexanucleotides for *Arabidopsis *as well as for *D. melanogaster *(Figure 3 and see Additional File 1). In the case of the TATAAA motif, peaks found for the two organism sets exceeded the average background distribution by 50-fold or more. Only the TATAAA element formed an analyzable peak in the dataset from *C. elegans*, but only in the ATG-rooted set (Figure 3). This might be explained either by the formation of multigene operons in *Caenorhabditis*, in which 15 % of all genes underlying transcriptional regulation are organized as polycistronic pre-mRNA [27,28] or by the existence of alternative TATA-like motifs with a degenerate nucleotide composition. Consequently, without this information the examined motifs can not be identified with significance.

To investigate the general occurrence of motifs carrying the same content of T and A mononucleotides, all 50 permutations of the TATAAA or TATATA hexamers were mapped in our reference datasets to examine if A/T-content alone could be detected by positional frequency disequilibrium (Table 2). Most of these T/A-rich hexanucleotides have regions in their respective distribution curves that differed by more than 4 times the standard deviation from their average background deviation depending on the analyzed dataset. Nearly all of the examined motifs were relatively enriched in the *A. thaliana *and *D. melanogaster *datasets rooted with the TSS as well as in the ATG-rooted set of *D. melanogaster *(Table 2). In the single dataset of *S. cerevisiae *rooted to the ATG, 33 motifs still matched our stringent criteria, while in the corresponding *A. thaliana *set only 15 TA-hexanucleotides differed from their background frequency significantly. We found 29 different TA-permutations in both *C. elegans *datasets which were more frequent than their respective background model (Table 2). This varying enrichment of T/A-rich motifs can possibly be explained by different single nucleotide compositions close to the TSS or the ATG [see Additional File 3]. One other reason for the differences found might be the number of contributing sequences per dataset, as pointed out before.

**Table 2:** Analysis of all possible permutations of the TATA-box elements TATAAA and TATATA in the promoter sequences of the four model organisms.

Element	At1500tss	At1500patg	Sc1500atg	Dm1500tss	Dm1500atg	Ce1500tss	Ce1500atg
TATAAA	95.9	11.2	15.0	49.6	10.1	5.0	30.1
TATATA	81.6	11.1	16.3	10.9	4.8	6.7	10.9
ATATAA	67.7	6.8	15.1	18.0	5.2	6.7	15.5
ATAAAT	52.0	4.9	8.3	12.2	6.0	4.7	5.9
ATATAT	43.7	4.2	10.1	10.1	6.4	-	4.7
TAAATA	41.4	4.0	8.1	10.8	7.6	6.1	5.1
TTATAT	27.0	-	7.2	9.7	4.7	5.7	8.1
TTTATA	22.9	-	6.8	8.6	5.4	5.2	5.3
TTATAA	22.5	-	6.2	8.7	6.3	4.8	4.2
TATTTA	19.5	4.1	7.6	9.6	6.8	4.3	6.4
AATATA	14.1	-	13.5	11.4	6.3	-	4.5
TATATT	13.1	-	8.5	10.2	6.9	5.5	8.5
TTTAAA	12.8	-	5.1	15.1	6.3	5.1	4.5
TATAAT	12.3	-	5.4	7.9	5.3	4.8	5.9
TTAAAA	12.1	-	7.9	12.7	7.5	-	4.2
ATTATA	11.4	-	9.0	8.1	5.6	-	-
ATTTAA	9.8	4.6	4.5	11.6	6.6	8.0	6.1
ATTTAT	9.7	-	6.0	8.0	5.2	4.4	7.6
TTAAAT	9.1	-	5.1	12.4	6.8	5.3	4.7
TAATAA	9.0	-	7.3	10.9	7.1	6.7	4.5
TAAAAT	8.8	-	4.5	13.0	6.6	6.5	4.6
ATTAAA	8.5	-	6.7	9.6	6.5	4.4	-
TAAATT	8.4	-	-	11.7	5.5	6.0	8.1
AAATAT	8.2	-	8.6	10.5	5.5	6.5	-
TATTAA	8.1	-	6.8	9.6	6.3	4.2	4.5
AATTAA	7.7	4.2	9.7	10.0	5.8	4.6	4.5
ATAATA	7.5	-	8.9	9.3	6.6	4.9	-
AATTTT	6.3	-	9.6	10.7	5.5	16.5	7.7
TTAATA	6.3	-	6.5	10.3	7.0	6.8	4.6
TTTTAA	6.2	-	6.0	11.8	6.6	5.9	4.9
ATTAAT	6.1	4.3	5.7	7.6	5.8	6.2	6.6
ATATTA	5.9	-	5.7	9.8	6.2	4.1	4.0
AATAAT	5.9	-	5.9	8.8	5.8	7.2	6.3
TTATTA	5.9	-	5.4	8.7	5.5	-	6.3
TTAATT	5.7	4.3	4.8	8.8	5.5	11.6	11.2
TAATTA	5.7	4.1	6.5	7.4	5.9	5.2	5.3
AAATTA	5.6	-	6.2	10.0	5.9	-	-
ATAATT	5.3	4.4	5.9	9.3	5.5	5.7	6.7
AAAATT	5.3	-	10.6	10.0	6.8	4.5	-
TTTAAT	5.1	4.0	4.7	9.8	7.3	6.7	8.4
ATTATT	5.0	-	6.3	9.5	6.2	5.2	9.3
AAATTT	4.9	-	8.9	9.5	6.8	9.3	6.0
TATTAT	4.8	-	7.3	8.2	5.6	4.7	5.6
AATATT	4.8	-	5.7	9.6	6.1	9.5	8.0
TAATAT	4.8	-	7.7	8.5	7.6	5.9	5.7
ATTTTA	4.7	-	5.3	9.8	7.3	4.7	7.3
AATTAT	4.6	-	4.6	8.0	5.5	5.2	5.4
ATATTT	4.3	-	7.8	9.9	6.3	8.0	8.5
TAATTT	4.3	-	5.1	11.1	6.0	14.3	11.5
AATTTA	4.3	4.1	-	9.7	7.4	5.7	7.2

Motif distribution curves were conducted for TATA-box like motifs and searched for frequency disequilibria that exceed a ≥ 4 fold *SD *from the average. The fold *SD *differences over the background frequency at the position of the highest peak are given for each of the permutations [see Additional File 10]. Background average and *SD *were calculated as described in the material and methods. The table is sorted according to the fold differences found in the *Arabidopsis *promoters.

However, many of these T/A-rich motifs exhibit significant enrichment, yet specific motifs have more significant tendencies than others, indicating a stronger positional bias. Among the most significant hexanucleotides in all organisms except for *C. elegans*, are the well known TATA-box elements TATAAA and TATATA (Table 2), which dominate the significance index. Additionally, the ATATAA-motif, which possibly overlaps with either of these TATA-box elements, was relatively enriched in the *S. cerevisiae*, *D. melanogaster *and *C. elegans *promoters. The TSS dataset for *C. elegans *did not reveal any of those motifs, but is rich in AATTTT or TAATTT (Table 2), which is consistent with its mononucleotide frequency profile (Additional File 4).

**Figure 4:**
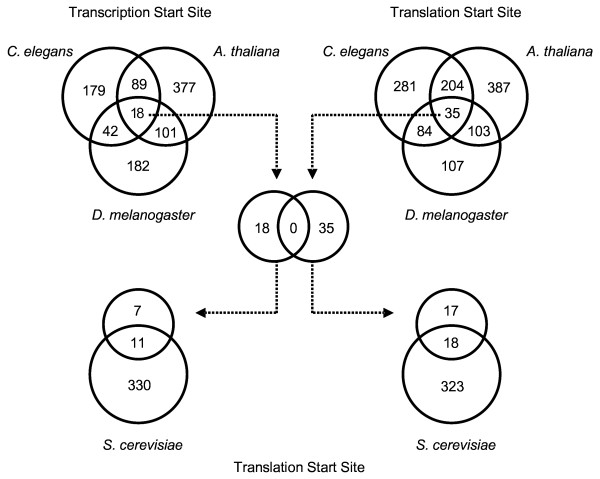
**Number of detected hexanucleotide motifs with a significant positional disequilibrium in different promoter datasets of the four model species**. Diagram of hexanucleotide motifs enriched in the promoters of *Arabidopsis*, *Caenorhabditis*, *Drosophila *and *Saccharomyces*. Significant motifs exhibiting a relative positional disequilibrium that differed ≥ 6 fold *SD *from the background average in promoter datasets spanning -1500 bp to -1 bp of the transcription start site (TSS) or the translation start codon ATG [see Additional File 6]. Raw values of all significant hexanucleotides are given in Additional File 11. Background averages and *SD *were calculated as described in the material and methods.

Hence, we can conclude that the motif analysis using frequency distribution maps from bulk sequence clearly identifies DNA-motifs containing various amounts of significant information. As we were able to validate previously characterized elements, we are convinced that this method of analysis gives important novel information on sequence data, as long as the amount of annotated genome sequence exceeds a certain minimal number of promoter sequences. We believe this threshold of a critical number of sequences will be specific for each organism.

### Hexanucleotide analysis with motif distribution curves in eukaryote promoters

We next took an unbiased approach to validate the quality of the datasets and the value of our approach. Therefore, we expanded our analysis and conducted motif distribution maps for all 4096 hexanucleotides. First, the three TSS rooted datasets from *Arabidopsis thaliana*, *Caenorhabditis elegans *and *Drosophila *melanogaster were examined independently of the ATG rooted sets. Initially, the resulting 24576 frequency distribution maps were inspected for motifs that exhibit behavior signatures implying functional significance near the TSS or the ATG. In general, the majority of hexanucleotide motifs show non-variant background behavior in sequence upstream of -600 bp from the respective roots. This proves that the majority of sequence variation, and hence putatively encoded information, is detectable near the TSS or the ATG. It is interesting to note that this observation had been made earlier [17,29,30].

In contrast to the previous, more direct analysis of the functional TATA-box sequences where 4 times the standard deviation was significant, we needed to obtain overrepresented hexanucleotide motifs with more than 6 times standard deviation from the average background distribution, based on the varying background frequencies. This can be explained by a large number of rare or infrequent motifs that incapacitate the identification of disequilibria based on the evaluation of background standard deviations alone. There are 585 hexanucleotide motifs relatively enriched in the *A. thaliana *TSS dataset, 343 in the set of *D. melanogaster *and 328 in that one of *C. elegans *(Figure 4). In the ATG rooted datasets 729 motifs were overrepresented for *A. thaliana*, 604 and 329 for *C. elegans *and *D. melanogaster*, respectively.

It is noteworthy, that the number of motifs differing in the two sets was higher in the set rooted with the ATG. While the number of significantly enriched hexanucleotides identified for *D. melanogaster *did not change drastically, there are 1.8 fold and 1.2 fold more motifs for *C. elegans *and *A. thaliana*, in the ATG datasets compared to the TSS datasets, respectively (Figure 4). This might be explained by the presence of more putatively functional motifs in the 5'UTR regions of *C. elegans *and *A. thaliana*, implying an important and possibly underestimated regulatory function for the UTRs in these species.

Although about 8 % to 10 % of the possible 4096 hexanucleotides were motifs relatively enriched over the background frequency, only 18 were found to be commonly overrepresented in a promoter specific manner in the three TSS sets of all three species. In the corresponding sets rooted for the translation start codon (ATG), 35 common hexanucleotides were overrepresented in the promoter of *A. thaliana*, *D. melanogaster *and *C. elegans*.

To our surprise, none of the 18 motifs found in the TSS sets overlapped with any of those 35 hexanucleotides enriched in the datasets of the ATG (Figure 4), which in turn suggests that the datasets may contain different non-coding regulatory sequences. To get a preliminary insight into the hexanucleotide composition of *S. cerevisiae *promoters, we compared the ATG rooted dataset with the motifs shared in the promoters of the three previous organisms. Of the 18 motifs relatively enriched in the TSS datasets, 11 were also significantly enriched in the set from *S. cerevisiae*. Of the 35 motifs overrepresented in the ATG rooted sets, 18 were more frequent in the yeast dataset (Figure 4).

Table 3 gives a closer insight into the 18 motifs overrepresented in the sets rooted with the TSS of the three organisms compared with the ATG rooted set of *S. cerevisiae*. Besides T/A-rich motifs like the previously studied TATA-like motifs, the hexanucleotides CATTTT and TCACAC differed most with respect to their background frequencies and constitute putative functional CREs conserved in the promoters of all eukaryotes. It is noteworthy that both motifs, CATTTT and TCACAC, have recently been found to have a function in several animal promoters, including humans [31,32].

**Table 3:** Motifs positioned at the transcription start site and shared between the four model organisms.

Element	At1500tss	Ce1500tss	Dm1500tss	Sc1500atg
TATATA	81.6	6.7	10.9	16.3
ATATAA	67.7	6.7	18.0	15.1
CTCTCT	43.3	6.2	8.1	4.2
TAAATA	41.4	6.1	10.8	8.1
GTATAT	25.8	8.0	11.2	7.8
ATTTAA	9.8	8.0	11.6	4.5
CTCTCG	9.4	8.1	6.2	-
GGCTAT	9.2	6.1	10.8	4.3
TAATAA	9.0	6.7	10.9	7.3
TAAAAT	8.8	6.5	13.0	4.5
AAATAT	8.2	6.5	10.5	8.6
TCACAC	7.1	6.3	15.3	5.2
CATTTT	6.5	14.8	6.4	7.6
AATTTT	6.3	16.5	10.7	9.6
TTAATA	6.3	6.8	10.3	6.5
TTATTT	6.3	10.3	10.2	8.4
TTTATT	6.1	8.9	10.4	7.6
ATTAAT	6.1	6.2	7.6	5.7

The standard deviation fold difference for the most significant peak is shown for each shared motif of the transcription start site datasets. Motif distribution curves were conducted and analyzed for all possible hexanucleotide motifs that have frequency disequilibria which exceeded ≥ 6 fold *SD *from the background average. Background average and *SD *were calculated as described in the material and methods. The table is sorted according to the fold differences found in the *Arabidopsis *promoters.

Similarly, among the 35 motifs more frequent in the ATG rooted datasets, the TATAAA motif of the TATA-box is enriched in the promoters of all organisms (Table 4). Four motifs contain the TCAG-motif present in the transcription initiation sites studied in the beginning of this work. Besides A-rich sequences, six hexanucleotides exhibiting a TCAA-core sequence pattern and two a palindromic TGCA-core motif, both of unknown function in eukaryotes, were found in the list of significant hexamers (Table 4).

**Table 4:** Motifs positioned at the translation start site and shared between the four model organisms.

Element	At1500atg	Ce1500atg	Dm1500atg	Sc1500atg
AGAAAA	23.8	12.6	7.0	13.9
AAAGAA	20.8	6.7	7.2	11.7
AACAAA	16.2	12.7	11.1	12.8
AGCAAA	15.6	13.0	15.6	13.0
ACAAAA	15.4	13.3	7.0	13.2
ACAACA	14.9	7.0	6.9	9.9
CAGAAA	11.7	33.9	7.2	5.5
CAAACA	11.3	6.6	6.9	9.5
TATAAA	11.2	30.1	10.1	15.0
ATCGAA	11.1	6.8	6.6	4.6
CCTATA	11.0	6.2	6.4	4.1
ATCAAA	11.0	15.0	12.4	5.9
CTCAAA	10.6	13.7	6.5	-
CAACAA	10.4	12.2	10.5	10.9
TCAGAA	10.3	47.7	7.0	-
CTATAA	10.3	11.0	7.8	5.9
TCGATT	9.5	6.2	6.2	-
CACAAA	9.5	15.0	15.1	8.2
TGCAGA	8.5	21.6	6.9	-
AACACA	8.3	6.8	11.1	13.9
AATCAA	8.3	11.9	13.6	6.6
TCAGCC	8.2	21.6	7.3	-
TCAAAC	8.0	9.8	6.9	-
CCCAAA	7.7	9.3	8.0	-
TTTCAG	7.6	121.5	7.3	4.7
TTGCAG	7.5	34.0	11.0	-
CAATCA	7.3	10.7	8.8	6.7
ATCAAC	7.2	11.2	12.5	4.5
CCAAAA	7.2	11.0	6.0	5.2
ACACAA	7.2	8.9	10.9	12.2
CTTCAA	7.0	11.6	7.5	-
TTCAGA	6.7	90.1	7.0	-
TCAAAA	6.7	14.1	6.4	5.3
ACCAAA	6.6	7.8	12.3	5.1
ACAGAA	6.2	9.9	10.2	5.7

The standard deviation fold difference for the most significant peak is shown for each shared motif of the translation start site datasets. Motif distribution curves were conducted and analyzed for all possible hexanucleotide motifs that have frequency disequilibria which exceeded ≥ 6 fold *SD *from the background average. Background average and *SD *were calculated as described in the material and methods. The table is sorted according to the fold differences found in the *Arabidopsis *promoters.

This analysis clearly identified several motifs that were not previously known to be conserved in promoters across organism kingdoms. Moreover, it proves the universal applicability of motif distribution curves for gathering useful positional information on promoter sequences.

### Motif distribution curves of known CREs from eukaryotes

As the TATA-box constitutes a conserved functional motif, we were interested to expand this study and discover whether other known functional *cis*-regulatory elements also form positional peaks that could be investigated by using motif distribution curves. Therefore, we retrieved functional CREs in sense and anti-sense orientation from literature or public databases for transcription factor binding sites.

We were able to analyze 450 motifs from *A. thaliana*, 51 for *S. cerevisiae *and 87 for both, *D. melanogaster *and *C. elegans*, (Table 5; see Additional File 5) in their respective promoter datasets with the idea to explore whether or not known functional CREs other than known core-promoter elements can be identified using motif distribution analysis. Here as well, we expected a high variability in frequency amongst the different CREs and, hence, applied a significance cutoff for a positional disequilibrium of ≥ 6 *SD *difference from the background average for our automated analysis. Rare motifs, with a background average of ≤ 1, were excluded. Taking into account that some of the boxes only function unidirectionally, for example the TATA-box, we scanned the promoter datasets for every CRE in both orientations. We found that at least 14,94 % of the known functional CREs do exhibit a significant positional disequilibrium matching our criteria (Table 5). The only exception was the TSS rooted set of *C. elegans *with only 8 % of positional enrichment of CREs. This might again be explained by the formation of multigene operons in *Caenorhabditis *[27] or the limited amount of promoter sequence. In total, we observe that depending on the organism or the dataset investigated 8 % to 50 % of the putatively functional CREs were enriched at a defined position (Table 5). Besides T- or A-rich CREs, we found a significant increase in frequency for several motifs with a higher GC-content in both the TSS and the ATG rooted datasets. In about half the cases of a CRE showing a positional enrichment in any of the datasets, it was orientation dependent (Table 5). Other known functional core-promoter elements like the DPE, BRE or the MTE [24,25,55] analyzed with datasets that extend over the transcription start sites could be detected only in Drosophila but not in the other species studied here (data not shown). Since the BRE and MTE are known to be enriched in TATA-less promoters [24], it may be worth performing a similar analysis with TATA-containing and TATA-less promoter datasets once more TSS information is available.

**Table 5:** Number of known functional *cis*-regulatory elements (CRE) that have positional disequilibria.

Dataset	Number of Cis-elements	number over 6-*SD*	sing.ori/both.ori/palindrome	percentage over 6-*SD*
Dm TSS	87	13	2/10/1	14,94%
Dm ATG	87	14	8/5/1	16,09%
	Dm shared	12	6/5/1	13,79%
At TSS	450	77	33/35/9	17,11%
At ATG	450	71	38/27/6	15,78%
	At shared	33	11/17/5	7,33%
Ce TSS	87	7	7/0/0	8,05%
Ce ATG	87	44	26/16/2	50,57%
	Ce shared	7	7/0/0	8,05%
Sc ATG	51	16	7/8/1	31,37%

*Cis*-elements [see Additional File 5] were retrieved and tested for positional disequilibrium as described in the text. CREs were analyzed for orientation bias (sing. ori = present in one orientation, both ori. = present in both orientations, or a palindrome). Percentage is the proportion of CREs relative to the respective total number of *cis*-elements investigated [see Additional File 12].

Although we have identified significantly enriched CREs with a positional disequilibrium on the basis of the peak height, valuable information is still retained in the frequency-distribution map of each motif, such as the breadth of enrichment.

**Figure 5:**
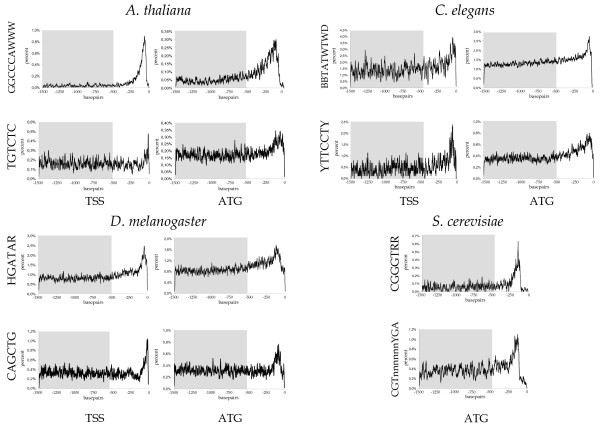
**Examples of frequency distribution curves of known *cis*-regulatory elements (CREs)**. Two randomly chosen examples for CREs that possess positional disequilibria were shown for each of the model organisms. Known functional *cis*-regulatory elements of the three kingdoms (animals, fungi and plants) were taken from Table 5. Relative number of motifs per site (in percent) was mapped to their respective position. The grey box indicates the region used to calculate the background average and its *SD*. For raw values of the illustrated examples see Additional File 13.

Since it is impossible to depict frequency distribution curves for all CREs, we have randomly chosen two per organism that do not represent known core-promoter elements from our list of significant CREs for presentation (Figure 5). In the following paragraphs, we describe these examples in more detail.

#### *A. thaliana*

The 450 CREs for Arabidopsis were derived from the Plant *cis*-acting regulatory DNA elements (PLACE) database [14,56]. The GGCCCAWWW motif was found in genes induced after decapitating the inflorescences and might be a CRE for wound stress response and the initiation of axillary bud outgrowth in Arabidopsis [33]. The TGTCTC motif is known as the DR5-element and represents the binding consensus for the Auxin Response Factor 1 (ARF1) found in promoters of early auxin responsive genes [34]. This CRE is used as an important reporter element in a synthetic promoter to trace developmental processes in which the phytohormone auxin is involved [35-37]. Interestingly, the synthetic promoter contains a multimeric repeat of the TGTCTC consensus at a similar position where we identified a relative enrichment of this motif in our distribution curves.

#### *S. cerevisiae*

The 51 CREs for yeast were derived from the promoter database SCPD [38,57]. The CGTnnnnnnYGA motif represents the binding consensus for ABF1 [39]. The second motif, CGGGTRR, constitutes the binding motif for the REB1 protein [40]. Interestingly, both proteins are multifunctional, site-specific DNA-binding proteins that act in concert in many biological processes and are both involved in preventing gene expression at the silencing mating-type locus [41,42]. A positional enrichment of the binding consensi for both transcription factors about 100 to 200 bp upstream the ATG is congruent with their function as general regulatory factors at the transcription initiation site [43].

Since no specific CRE databases exist for *C. elegans *and *D. melanogaster*, we retrieved 87 CREs primarily from literature and textbook sources [see Additional File 5].

#### *C. elegans*

The BBTATWTWD consensus was identified in a phylogenetic footprint approach to form a regulatory module, which confers a muscle-specific gene expression [44]. The YTTCCTY motif is the binding consensus of ETS-transcription factors [45], which are associated with several physiological and pathological processes. They contribute to the regulation of gene expression during the maturation of hematopoietic cell lineages and in tumor cell growth

#### *D. melanogaster*

The CAGCTG motif is known as the palindromic GC-box and represents a binding consensus conserved in 502 RNA polymerase II promoter regions [46]. The HGATAR motif was chosen as it was relatively enriched in *Drosophila *promoters, although it was described as a binding site for mesoderm specific MED1 GATA-factor in *C. elegans *[47]. This conforms to the idea that some *cis*-elements are conserved over large evolutionary distances and that our approach can identify such putatively functional motifs in eukaryote genome comparison approaches. For example, GAGA-like elements were first described in *Drosophila *and have recently been found to be functional elements in plants as well [48,49].

## Conclusion

It was proposed that the TATA-box was the only functional motif shared in the promoters between eukaryotes of different kingdoms [11] based solely on a frequency derived background model. Instead, our analysis has shown several highly conserved motifs besides TATA-box like patterns common in the promoter sequences flanking the TSS or the translation start codon ATG even amongst distantly related species.

Genome wide promoter analysis with frequency distribution maps expands our knowledge of the core promoter and the majority of known functional CREs. It has long been known that the mere presence of an element does not necessarily result in its functionality with respect to transcriptional regulation [5,6]. Hence, existing database information on CREs is valuable resource, i.e the PLACE collection of plant CREs [14] or the SCPD for yeast [38], but without positional information one could not reach any definite conclusion as to the significance of its putative functionality. With the information on positional enrichment of a motif or a known functional CRE it is possible to get a more detailed insight into where an element appears to function. This in turn might accelerate the in depth examination of regulatory sequences in the laboratory.

It has been shown that essential *cis*-regulatory modules exist in *Drosophila melanogaster *that control gene expression from a distance of several kilobases [50]. Nonetheless, we identify functionally important motifs to be located close to the TSS or the ATG. Whether such modules effect transcription via chromatin remodeling and nucleosome positioning, rather than a direct influence on the transcriptional machinery, remains to be examined.

On the basis of the average position of the TATA-box in the ATG rooted dataset, it also became evident that the 5'UTRs of different species can be of similar sizes, as was published before [see Additional File 2, [23]]. Moreover, some sequence pattern might be positionally enriched in the 5'UTR, implying an important and possibly underestimated regulatory function, but information on these kinds of elements is rare and needs more experimental investigation.

We concentrated on motifs which have maxima. But during this work, it became evident that motifs exist, which exhibit significant positional minima in the frequency distribution curves. Perhaps a detailed investigation of them could also contribute to our current understanding of gene regulation.

As our analysis was carried out on regular personal computers and the scripts were written in the Visual Basic script language, the analysis can be performed inexpensively and freely adopted or modified to suit specific needs. The universal applicability of our method and the simplicity of the background model used is a clear advantage of the presented approach. The use of motif distribution curves will greatly enhance the quality of the identification of novel promoter motifs, as many of the known functional CREs appear to be relatively enriched at defined sites near the core promoter region.

## Methods

### Motif mapper

The vast majority of the developed scripts mentioned in this work have been deposited in the Motif Mapper open-source script collection first released online in 2004 [58]. The Motif Mapper package is routinely maintained and up-dated at its web site, so that only the current version is available. A history file is available, documenting all changes and modifications made to the script code and package composition. Script version numbers mentioned refer to the individual script version number regardless of the package version which can be found in the beginning of each script file. Additionally, any older versions that have been replaced during package updates can be readily obtained upon request. Detailed descriptions accompany the package at its webpage.

### Extraction and alignment of sequences

The GenBank DNA sequence flat-files were downloaded from the Entrez Plant Genomes Central at NCBI [59] for *Arabidopsis thaliana *[GenBank: NC_003070.5, GenBank: NC_003071.3, GenBank: NC_003074.4, GenBank: NC_003075.3, GenBank: NC_003076.4]; *Drosophila melanogaster *[GenBank: NC_004353.2.gb, GenBank: NC_004354.2.gb, GenBank: NT_033777.2.gb, GenBank: NT_033779.3.gb, GenBank: NT_037436.2.gb]; *Saccharomyces cerevisiae *[GenBank: NC_001133.5, GenBank: NC_001134.7, GenBank: NC_001135.3, GenBank: NC_001136.6, GenBank: NC_001137.2, GenBank: NC_001138.4, GenBank: NC_001139.5, GenBank: NC_001140.4, GenBank: NC_001141.1, GenBank: NC_001142.5, GenBank: NC_001143.5, GenBank: NC_001144.3, GenBank: NC_001145.2, GenBank: NC_001146.3, GenBank: NC_001147.4]; *Caenorhabditis elegans *[GenBank: NC_003279.2.gbk, GenBank: NC_003280.2.gbk, GenBank: NC_003281.3.gbk, GenBank: NC_003282.2.gbk, GenBank: NC_003283.3.gbk, GenBank: NC_003284.3.gbk]. Each chromosome sequence was extracted to an additional file using the SeqGBComplie v1.2 script.

Upstream regions rooted to the annotated transcription start site (TSS) or the translation start site (ATG) were extracted and compiled to FASTA formatted text with the aGenBankSQL script v3.3.4, using the default code modification suggested in the Motif Mapper website when extracting the TSS rooted datasets. This code modification only extracts promoters from genes that have an annotated or predicted 5'UTR. In cases where several TSS or ATG are annotated, the routines were adjusted in such a way that only the first annotation was recognized resulting in only one extracted sequence contributing to the promoter datasets. For each genome, the individual pseudo-molecule promoter datasets (i.e. each FASTA file per chromosome) were assembled into one file using the TxtFileFuse v1.2 script. Thereafter, frequency distribution curves were created for each motif by first combining the established promoter datasets with the StackerNtrimmer v.3.2.2 script to create the input files needed for the enumerating script REG_Pro_Point_Mapper v3.4.0. The number of extracted promoters is: At ATG 26299; At TSS 16997; Dm ATG 10671; Dm TSS 7953; Ce ATG 19179; Ce TSS 885; and Sc ATG 5361. A flowchart of the extraction and analysis is given as Additional File 4.

### Frequency analysis of motifs

The REG_Pro_Point_Mapper algorithm identifies non-overlapping motifs in each promoter from 5' to 3' orientation and records the position of the motif in its respective position in the promoter. Each sequence is searched independently and hits at the same position in two promoters are added together. A hexanucleotide hit is recorded as 111111. Two overlapping hexamer motifs at identical positions in two different sequences result in 222222; hence, hexanucleotide motifs which overlap in their respective position by only one nucleotide in two different promoters are recorded as 11111211111. Using this approach, we eliminated the need for a transformation to catch elements that have partial overlaps. Furthermore, simple smoothing algorithms tend to destroy the positional information in biological sequences (unpublished data). This is particularly evident when a motif, e.g. the translation start codon "ATG", is tightly positioned with respect to a biologically functional location within the DNA strand. Hence, models that average over a word size larger than the motif itself lose this positional information. Therefore, no smoothing operations have been applied to the data.

### Frequency distribution curves

Frequency distribution curves were conducted on the text-file ".psum" output of REG_Pro_Point_Mapper v3.4.0 with regular spread sheet analysis programs. For each rooted dataset the raw number of motifs was plotted in respect to their exact position. As all sets used in this analysis were either rooted to the transcription start site (TSS) or the translation initiation codon ATG, all curves were drawn with the root pointing to the right.

### Detection of significant change in positional disequilibria

Automatically evaluated motif distribution curves were searched for frequency disequilibria that exceed a defined fold standard deviation (*SD*) from the average occurrence. We used the first 1000 bps (-1494 bp to -494 bp) from the 1500 bp promoter sets to derive the background average, or the entire data set for all smaller datasets. The average background frequency constitutes the background model. We then calculated the standard deviation for each motif by using the following equation:

SD=∑(xi−x¯)2(n−1)
 MathType@MTEF@5@5@+=feaafiart1ev1aaatCvAUfKttLearuWrP9MDH5MBPbIqV92AaeXatLxBI9gBaebbnrfifHhDYfgasaacH8akY=wiFfYdH8Gipec8Eeeu0xXdbba9frFj0=OqFfea0dXdd9vqai=hGuQ8kuc9pgc9s8qqaq=dirpe0xb9q8qiLsFr0=vr0=vr0dc8meaabaqaciaacaGaaeqabaqabeGadaaakeaacqWGtbWucqWGebarcqGH9aqpdaGcaaqaamaalaaabaWaaabqaeaacqGGOaakcqWG4baEdaWgaaWcbaGaemyAaKgabeaakiabgkHiTiqbdIha4zaaraGaeiykaKYaaWbaaSqabeaacqaIYaGmaaaabeqab0GaeyyeIuoaaOqaaiabcIcaOiabd6gaUjabgkHiTiabigdaXiabcMcaPaaaaSqabaaaaa@3F7D@

where *x*_*i *_is the number of counts at position *i*, x¯
 MathType@MTEF@5@5@+=feaafiart1ev1aaatCvAUfKttLearuWrP9MDH5MBPbIqV92AaeXatLxBI9gBaebbnrfifHhDYfgasaacH8akY=wiFfYdH8Gipec8Eeeu0xXdbba9frFj0=OqFfea0dXdd9vqai=hGuQ8kuc9pgc9s8qqaq=dirpe0xb9q8qiLsFr0=vr0=vr0dc8meaabaqaciaacaGaaeqabaqabeGadaaakeaacuWG4baEgaqeaaaa@2E3D@ is the average from the background model and *n *is the number of promoters in the dataset.

Assuming that motifs are equally distributed apart from any positional disequilibrium, we decided to use a simple significance cutoff frequency according to Arkhipova [20], defined as ≥ 4 *SD *from the average. Significant deviances from this average by 4 standard deviations were then used as a screening parameter to identify peaks and valleys. For evaluation on the curves for the 4096 hexanucleotides and known CREs, we introduced a higher cutoff frequency at ≥ 6 *SD *than the average of the background model. As certain motifs differ significantly in their frequencies near the transcription or translation start sites, but are not strictly position dependent (e.g. T/A rich motifs), the length of a significant stretch is recorded as a "gap". The script pSUMscan v.3.4.0 was written to automatically catalogue the peaks and valleys and gap data with the default set to 4 standard deviations (*SD*) above or below the background average; gaps were set to a default length of 25 bp. This entire automated analysis performed by the pSUMscan script can also be manually done in common spread sheet analysis programs.

### *Cis*-regulatory Elements (CREs)

Known CREs were retrieved using the well maintained databases PLACE ([56]; 450 elements) for plant motifs and SCPD ([57]; 51 elements) for motifs from *Saccharomyces*. In order to gather CREs for *Drosophila *or *Caenorhabditis*, literature describing various eukaryotic *cis*-elements was collected and catalogued into a list of 87 elements. This list carries the original description of the *cis*-element, the IUPAC conversion used in this work and the original reference source describing the element. Apart from palindromes, we conducted motif distribution curves for sense and the antisense orientation of the motifs independently. The list of cis-elements used from PLACE, SCPD and other eukaryotes is found in Additional File 5.

### Motif permutations

All possible permutations of the hexamers TATAAA and TATATA for each nucleotide were generated with the MAIN_AllCombinations v.3.4.1 script. Antisense motifs were generated by the MAIN_List_w_RevComps v.1.2 routine and all redundant motifs were removed with the script MAIN_ClearRedundancy v.1.2.

### Shared motifs

Shared motifs were identified by conducting text file lists of overrepresented motifs for each dataset. The list content was compared using the SetGrouping script to identify shared motifs in both lists. Details can be found in Additional File 6.

## Abbreviations

TSS, transcription start site; ATG, translation start site; CRE, *c*is-regulatory element; *SD*, Standard Deviation; *At*, Arabidopsis thaliana; *Sc Saccharomyces cerevisiae*; *Dm*, *Drosophila melanogaster*; *Ce*, *Caenorhabditis elegans*.

## Authors' contributions

All authors have read and approved the manuscript.

DW and KS initiated the development of frequency-distribution analysis of *cis*-element distribution, KS and KB developed algorithms for analysis, KB programmed the Motif Mapper open source package, KH provided continuous support and research space for completion of this work.

## Supplementary Material

Additional File 1 Distribution curves of the TATA-box motifs TATAAA and TATATA. The motif distribution curves of the TATA-box hexanucleotides TATAAA and TATATA were constructed on automatically assembled datasets of the *Arabidopsis*, *Caenorhabditis*, *Drosophila *and *Saccharomyces *genome sequences. Relative number of motifs per site (in percent) was mapped to their respective position [see Additional file 9]. The grey box indicates the region used to calculate the background average and its *SD*. Click here for file

Additional File 2 Size distribution of 5' UTRs. The 5'UTR lengths from each TSS dataset were plotted against their relative frequency. Click here for file

Additional File 3 Mononucleotide frequencies at the transition initiation sites TSS and ATG. The motif distribution curves of the four nucleotides were constructed on automatically assembled datasets of the *Arabidopsis*, *Caenorhabditis*, *Drosophila *and *Saccharomyces *genome sequences. Relative number of nucleotides per site (in percent) was mapped to their respective position. Click here for file

Additional File 4 Motif Mapper analysis flowchart. A flow chart of the sequence extraction and analysis procedure using Motif Mapper. Click here for file

Additional File 5 List of *cis*-regulatory elements and references. List of all *cis*-regulatory elements, their IUPAC version used in this work and their references. Click here for file

Additional File 6 Hexanucleotides positioned at the transcription or the translation start site and shared between the four model organisms. The standard deviation fold difference for the most significant peak is shown for each shared motif of the transcription or the translation start site datasets. Motif distribution curves were conducted and analyzed for all possible hexanucleotide motifs that have frequency disequilibria which exceeded ≥ 6 fold *SD *from the background average. Click here for file

Additional File 7 Raw data for Table 1. Contains the .psum-data for all of the motifs used in the *D. melanogaster *dataset comparisons between Ohler et al., (2002, [21]) and GenBank (2005). Click here for file

Additional File 8 Peak analysis for Table 1. Peak analysis of 4-mer motifs used for *D. melanogaster *dataset comparisons between Ohler et al., (2002, [21]) and GenBank (2005), based on the raw information [see Additional file 7]. Click here for file

Additional File 9 TATA-hexanucleotide comparison curves. Contains the .psum files and graphs for the elements TATATA and TATAAA for all genomes analyzed. Click here for file

Additional File 10 Peak analysis for the TATA-hexanucleotide comparison. Peak analysis files used for TATATA and TATAAA permutation analysis, including the summary table. Click here for file

Additional File 11 All significantly enriched hexanucleotides. Peak analysis of all hexanucleotides that have *SD *fold differences of 4 or more for each dataset, including summary file. Click here for file

Additional File 12 Analysis of all known functional *cis*-regulatory elements. Peak analysis of all Cis-regulatory elements (CREs) that have *SD *fold differences of 4 or more for each dataset, including summary file. Click here for file

Additional File 13 Raw data and graphs for Figure 5. Raw data on the two randomly chosen examples for CREs that possess positional disequilibria shown in Figure 5. Click here for file
