# Supplementary material for: Cis-motifs upstream of the transcription and translation initiation sites are effectively revealed by their positional disequilibrium in eukaryote genomes using frequency distribution curves
Source: BMC Bioinformatics. 2006 Nov 30;7:522. doi: 10.1186/1471-2105-7-522 (PMC1698937; doi:10.1186/1471-2105-7-522)
Supplement: Additional File 1 — Distribution curves of the TATA-box motifs TATAAA and TATATA. The motif distribution curves of the TATA-box hexanucleotides TATAAA and TATATA were constructed on automatically assembled datasets of the Arabidopsis, Caenorhabditis, Drosophila and Saccharomyces genome sequences. Relative number of motifs per site (in percent) was mapped to their respective position [see Additional file 9]. The grey box indicates the region used to calculate the background average and its SD. [file 1471-2105-7-522-S1.pdf]

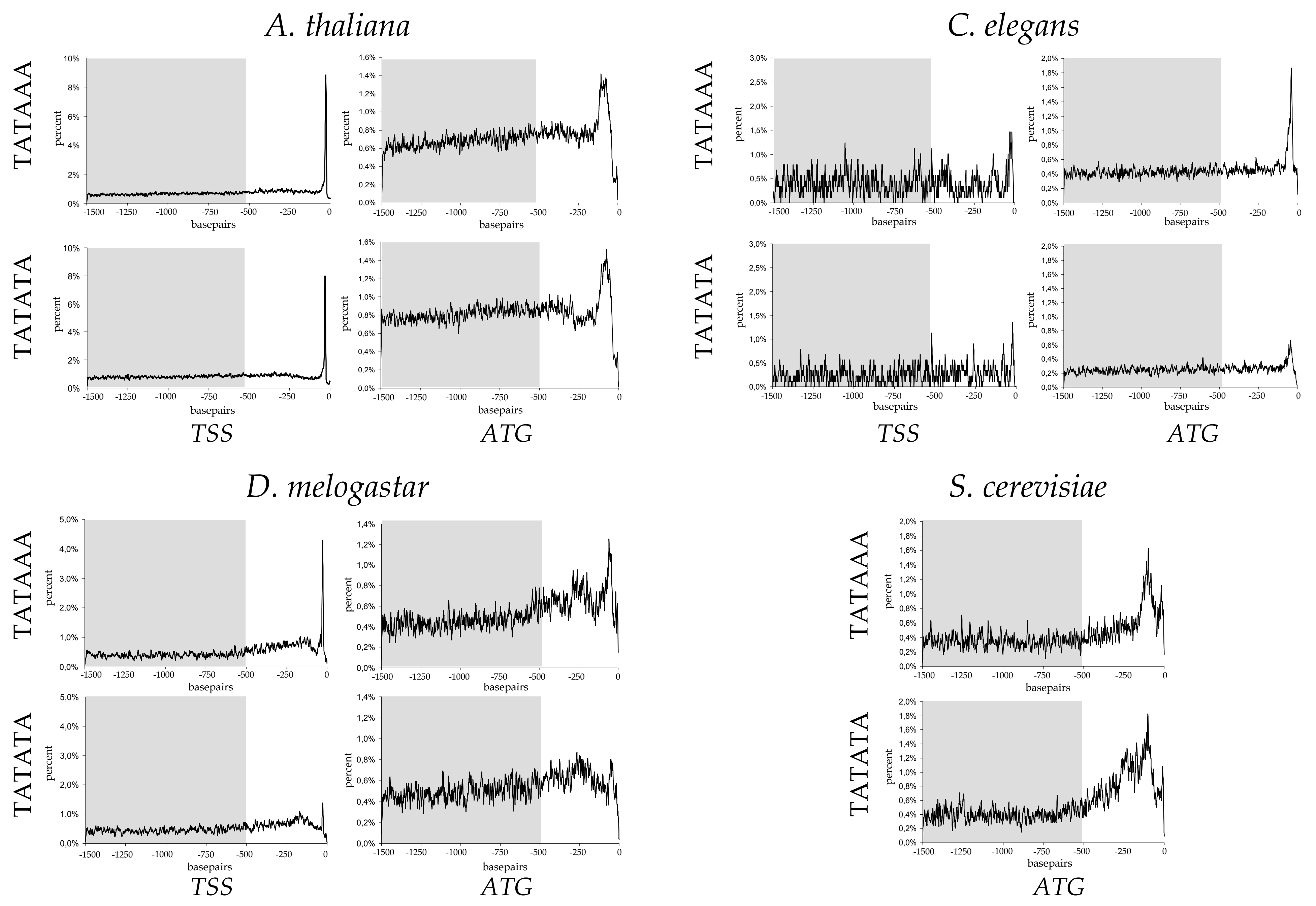

A. thaliana
C. elegans
D. melogastar
S. cerevisiae
TATAAA
TATATA
percent
basepairs
TSS
ATG
