# Supplementary figures and images for: Cis-motifs upstream of the transcription and translation initiation sites are effectively revealed by their positional disequilibrium in eukaryote genomes using frequency distribution curves
Source: BMC Bioinformatics. 2006 Nov 30;7:522. doi: 10.1186/1471-2105-7-522 (PMC1698937; doi:10.1186/1471-2105-7-522)

# Size distribution of 5'UTRs

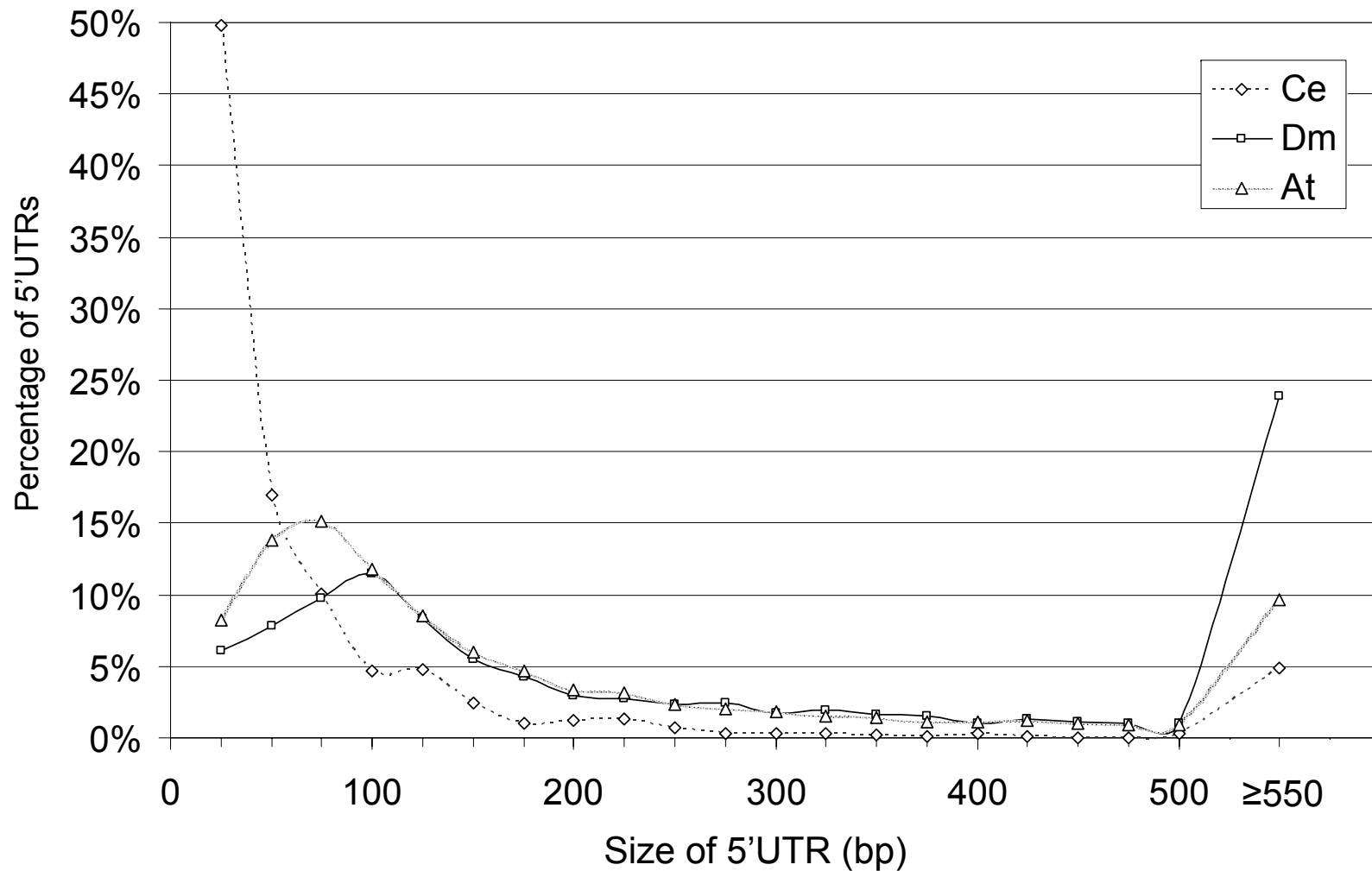

Supplement: Additional File 2 — Size distribution of 5' UTRs. The 5'UTR lengths from each TSS dataset were plotted against their relative frequency. [file 1471-2105-7-522-S2.pdf]

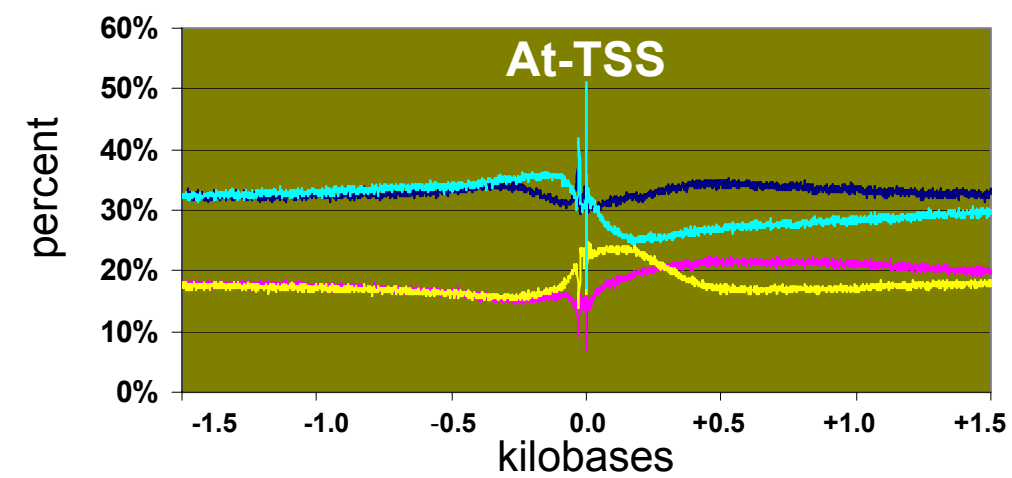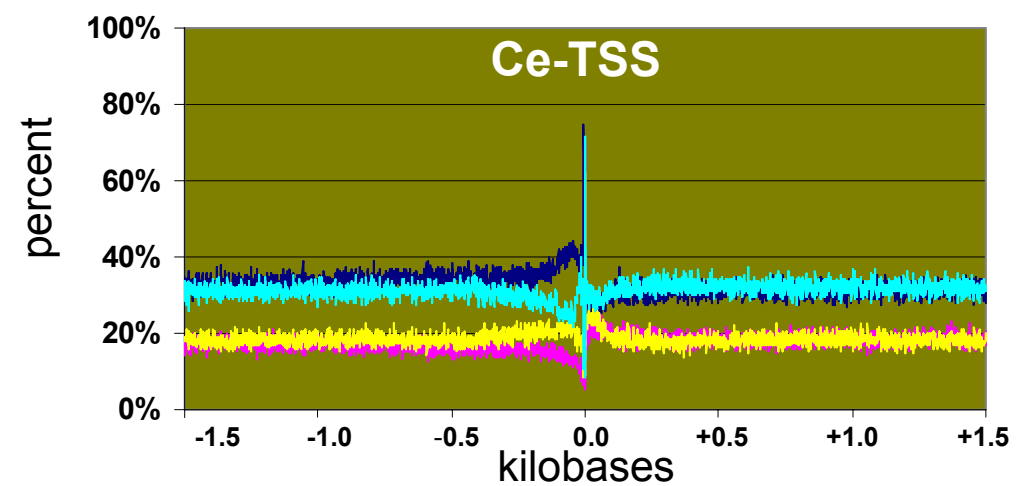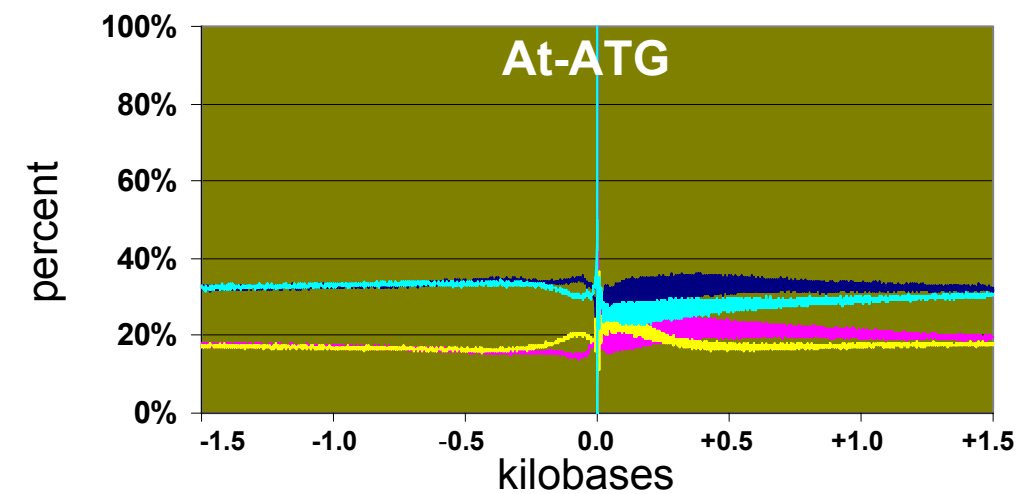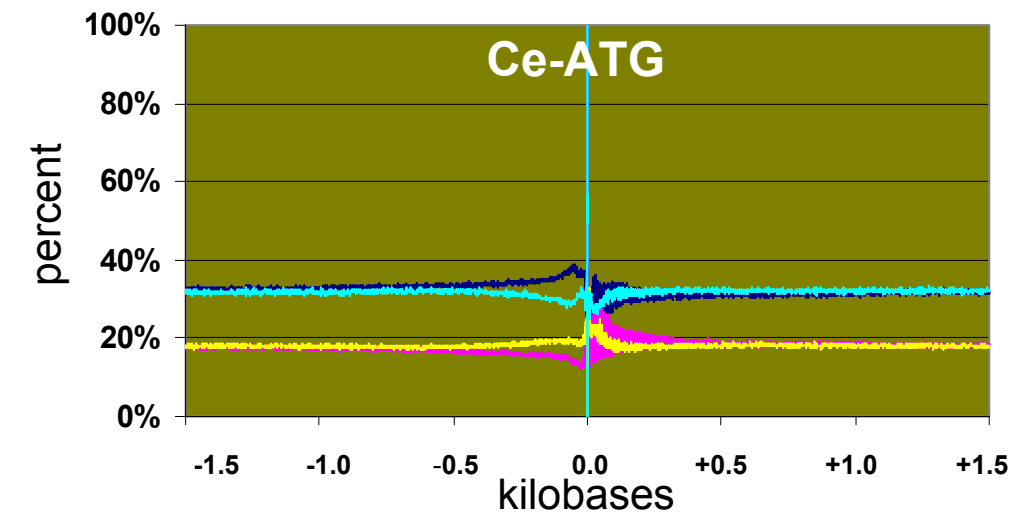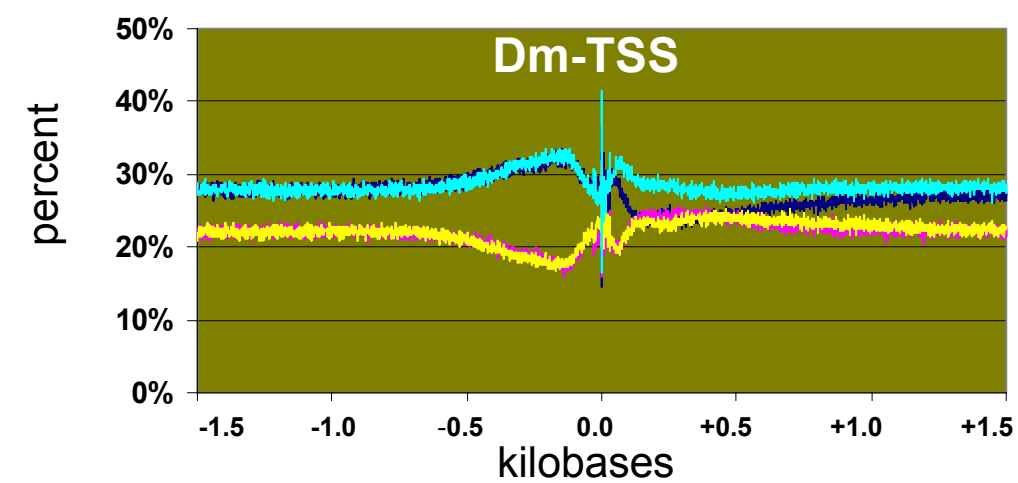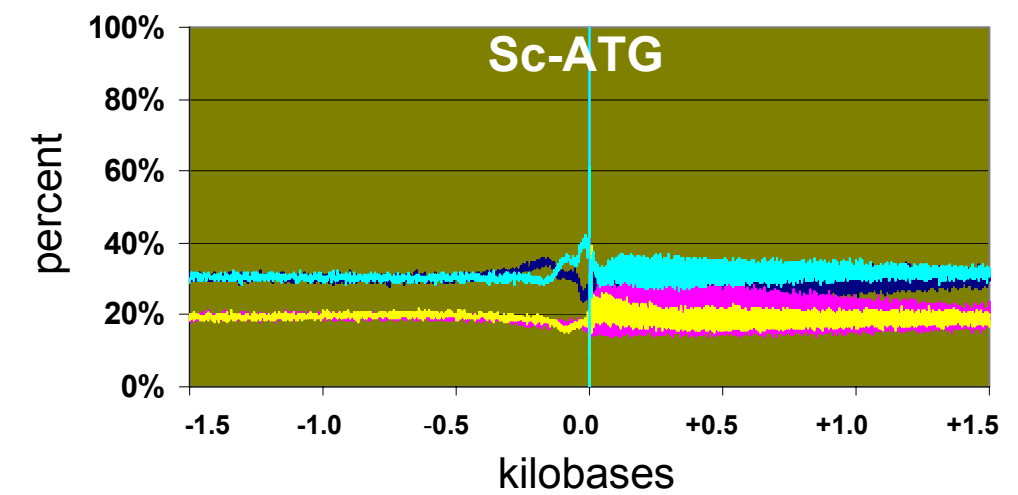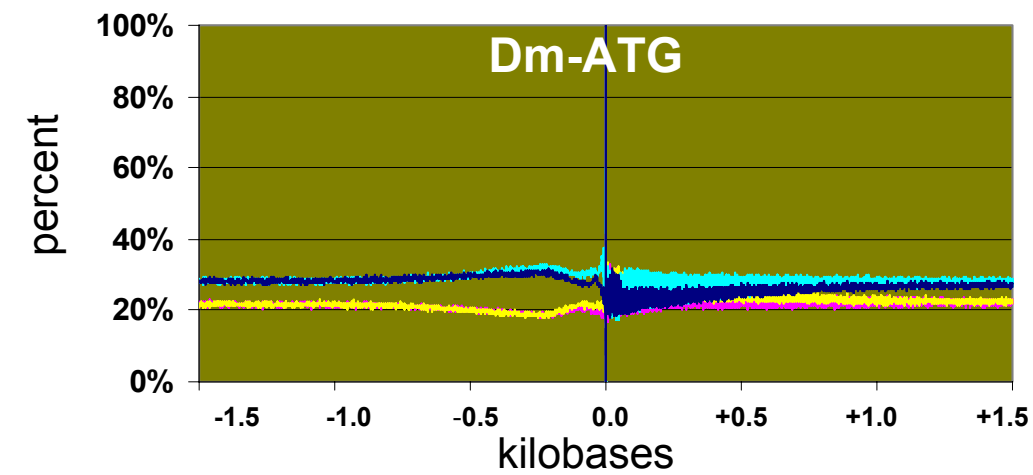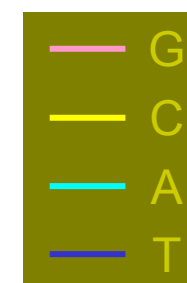

Supplement: Additional File 3 — Mononucleotide frequencies at the transition initiation sites TSS and ATG. The motif distribution curves of the four nucleotides were constructed on automatically assembled datasets of the Arabidopsis, Caenorhabditis, Drosophila and Saccharomyces genome sequences. Relative number of nucleotides per site (in percent) was mapped to their respective position. [file 1471-2105-7-522-S3.pdf]
