# Supplementary material for: Cis-motifs upstream of the transcription and translation initiation sites are effectively revealed by their positional disequilibrium in eukaryote genomes using frequency distribution curves
Source: BMC Bioinformatics. 2006 Nov 30;7:522. doi: 10.1186/1471-2105-7-522 (PMC1698937; doi:10.1186/1471-2105-7-522)
Supplement: Additional File 4 — Motif Mapper analysis flowchart. A flow chart of the sequence extraction and analysis procedure using Motif Mapper. [file 1471-2105-7-522-S4.pdf]

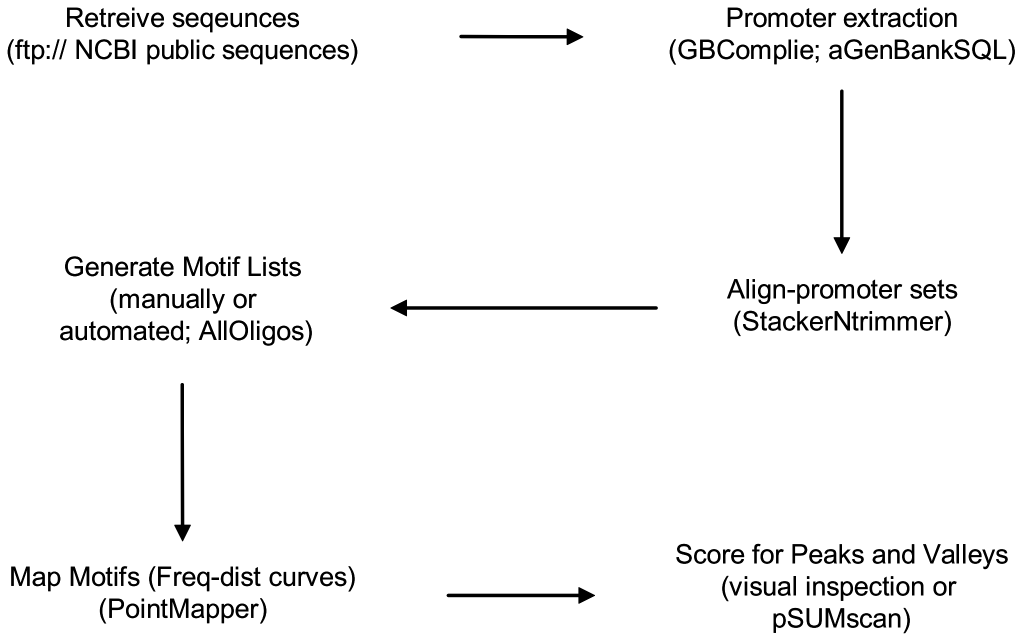
Retreive seqeunces
(ftp:// NCBI public sequences)
Promoter extraction
(GBComplie; aGenBankSQL)
Align-promoter sets
(StackerNtrimmer)
Generate Motif Lists
(manually or
automated; AllOligos)
Map Motifs (Freq-dist curves)
(PointMapper)
Score for Peaks and Valleys
(visual inspection or
pSUMscan)
